# The effect of physical loading on calcaneus quantitative ultrasound measurement: a cross-section study

**DOI:** 10.1186/1471-2474-13-70

**Published:** 2012-05-14

**Authors:** Cheng-Rui Liu, Hai-Jun Niu, Fang Pu, Ling Wang, Lian-Wen Sun, Yu-Bo Fan, De-Yu Li

**Affiliations:** 1 Key Laboratory for Biomechanics and Mechanobiology of Ministry of Education, School of Biological Science and Medical Engineering, Beihang University, XueYuan Road No.37, Beijing, 100191, People’s Republic of China

**Keywords:** Quantitative ultrasound, Osteoporosis, Physical loading, Menopause

## Abstract

**Background:**

Physical loading leads to a deformation of bone microstructure and may influence quantitative ultrasound (QUS) parameters. This study aims at evaluating the effect of physical loading on bone QUS measurement, and further, on the potential of diagnosing osteoporosis using QUS method under physical loading condition.

**Methods:**

16 healthy young females (control group) and 45 postmenopausal women (divided into 3 groups according to the years since menopause (YSM)) were studied. QUS parameters were measured at calcaneus under self-weight loading (standing) and no loading (sitting) conditions. Weight-normalized QUS parameter (QUS parameter measured under loading condition divided by the weight of the subject) was proposed to evaluate the influence of loading. T-test, One-Way analysis of variance (one way ANOVA) and receiver operating characteristic (ROC) analysis were applied for analysis.

**Results:**

In QUS parameters, mainly normalized broadband ultrasound attenuation (nBUA), measured with loading significantly differed from those measured without loading (*p* < 0.05). The relative changes of weight-normalized QUS parameters on postmenopausal women with respect to premenopausal women under loading condition were larger than those on traditional QUS parameters measured without loading. In ROC analysis, weight-normalized QUS parameters showed their stronger discriminatory ability for menopause.

**Conclusions:**

Physical loading substantially influenced bone QUS measurement (mainly nBUA). Weight-normalized QUS parameters can discriminate menopause more effectively. By considering the high relationship between menopause and osteoporosis, an inference was drawn that adding physical loading during measurement may be a probable way to improve the QUS based osteoporosis diagnosis.

## Background

It has been a long time since osteoporosis was recognized as a major public health problem, with enormous social and economic impact [1]. Currently, the best validated method for osteoporosis diagnosis is dual X-ray absorptiometry (DXA) [2,3]. However, this method is expensive and requires skilled radiographers. At the same time, the bone mineral density (BMD) measured by DXA only provides information on bone mass but contains little information on the microstructure and mechanical properties of the bone which are also important factors of osteoporosis [4,5]. In recent years, quantitative ultrasound (QUS) has been recognized as a novel technique for osteoporosis diagnosis [6]. Although the sensitivity of QUS is not enough for a clinical osteoporosis diagnosis [7], it has other advantages such as low cost, with no radiation, and most importantly, providing comprehensive information on both bone mass and the microstructure of the bone [8-10]. As a result, QUS has been recommended as an effective screening technique for digging out osteoporosis suspects from large populations [7].

In previous studies, QUS measurement was normally performed on bone specimens without physical loading (sitting) [7-12]. However, in situ human bones, especially those composing the axial and appendicular skeleton, are subjected to repetitive, cyclic loading during routine daily activities. Experimental results showed that the microstructure of the bone deformed and mechanical properties of the bone changed significantly under physical loading [13,14]. On the other hand, the propagation of ultrasound wave is influenced not only by bone mass but also by bone microstructure and material properties [15-18]. As a result, it is possible that the physical loading exerted on bone will not change BMD (due to the mineral content of the bone is not changed) but the QUS parameters (due to the microstructure of the bone deformed and mechanical properties of the bone changed under loading condition). In addition, these changes of QUS parameters should be more on bones with osteoporosis, since bones with osteoporosis are easier to distort than normal bones under the same loading condition. Then we proposed the hypothesis that more distortions on the microstructures of osteoporosis bones under loading condition may help to discriminate the osteoporosis bones more effectively from normal bones using bone QUS measurement (i.e. adding loading may help improve the sensitivity of QUS based osteoporosis diagnosis). Since low sensitivity (i.e. overlapping of QUS parameters measured from osteoporosis patients and normal people) is currently the most significant problem in QUS based osteoporosis diagnosis [7], if this hypothesis is valid, the findings will help to find a possible way to overcome this significant shortcoming in bone QUS osteoporosis diagnosis. Based on our hypothesis, an experiment was conducted in this preliminary study in order to verify: 1) whether bone QUS parameters measured under loading condition differ from those measured without loading; 2) if the answer to 1) is “yes”, is it possible that exerting loading during ultrasound measurement can provide more useful information to osteoporosis diagnosis, or on the other word, to potentially improve the osteoporosis diagnosis based on QUS?

In this study, QUS measurements were conducted at calcaneus on pre and postmenopausal women under both loading (standing) and no loading (sitting) conditions. “Standing” means that the subject’s self-weight is used as loading exerted on calcaneus during measurements. By considering the consistent and high relationship between menopause and osteoporosis [4,19,20], the postmenopausal women were considered as approximations to osteoporosis or osteopenia. To our best knowledge, this is the first study which investigates the effect of loading on the bone QUS measurement.

## Methods

### Materials

In total, 61 females were enrolled, including 16 healthy females aged below 30 years and 45 postmenopausal women aged from 50 to 77 years. All subjects were recruited from the same region of the country and were divided into four groups, one control group (group 0, n = 16, age: 23.4 ± 1.05 years, weight: 50.1 ± 4.9 kg) and three testing groups (postmenopausal women) according to the years since menopause (YSM): 1 ≤ YSM ≤ 10 for group1 (n = 12, age: 56.2 ± 2.8 years, weight: 63.1 ± 8.7 kg, YSM: 5.0 ± 2.0 years), 11 ≤ YSM ≤ 20 for group 2 (n = 18, age: 63.4 ± 4.2 years, weight: 62.8 ± 9.7 kg, YSM: 14.5 ± 2.9 years) and 21 ≤ YSM ≤ 30 for group 3 (n = 15, age: 73.2 ± 2.9 years, weight: 59.7 ± 8.9 kg, YSM: 25.7 ± 2.8 years). Informed consents were obtained from all participants before the study. This study was approved by the Beihang university ethics committee.

### QUS measurement

**Figure 1:**
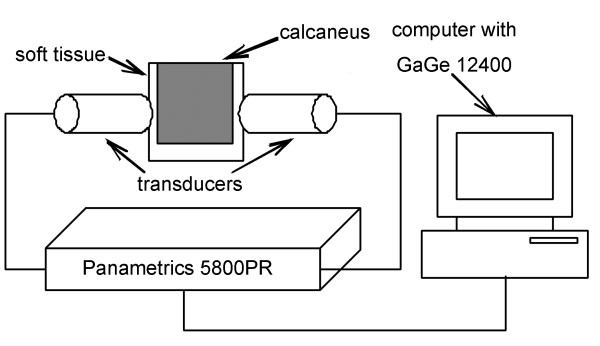
**Block diagram of QUS measurement system.** Two ultrasound transducers are fixed coaxially on both sides of the calcaneus and are coupled to the skin through coupling gel. Ultrasonic pulse is generated from one transducer, transmitting through the calcaneus and detected by another transducer. The detected signal is amplified by 5800 PR and output to GaGe CompuScope 12400, where the signal is sampled and converted to digital signal for further analysis.

QUS parameters were measured on all subjects at calcaneus with self-weight loading (standing) and without loading (sitting). The measurement was performed using a Panametrics 5800PR ultrasonic pulser-receiver with a pair of V303-SU unfocused transducers (Olympus NDT, Waltham, MA USA). The central frequency and element diameter of the transducers were 1 MHz and 0.5 inch, respectively. During measurement, the two transducers were fixed coaxially on both sides of the calcaneus by a caliper and were coupled to the skin through coupling gel. The received signal was then collected by a PCI bus digitizer card GaGe CompuScope 12400 (GaGe Applied Technologies, Lockport, IL USA), sampled and converted to digital signal for analysis. Figure 1 shows the block diagram of our QUS measurement system. After the measurements at calcaneus, with the distance between two transducers unchanged, the ultrasound signal transmitting in water was measured as reference signal. All the above measurements were repeated 5 times for each subject.

### Calculation of QUS parameters

QUS parameters were calculated using MATLAB 7.0 (MathWorks, Natick, MA USA). Since the thicknesses of the skin and soft tissue covering on both sides of the calcaneus were very small and they became even smaller when these tissues were pressed by the transducers, it was assumed that the width of the calcaneus was equal to the distance between two transducers during the measurement. The speed of sound (SOS, m·s^-1^) was calculated by:

(1) $$SOS=\frac{l}{TOF}\text{,}$$

where ‘*l*’ is the width of the calcaneus and ‘*TOF*’ is the time of flight, defined as the time when the signal firstly reaches a fixed threshold which is experimentally determined prior to experiment as a value just above the peak of noise. For the calculation of broadband ultrasound attenuation (BUA, dB·MHz^-1^), the spectrum of the transmitting signal in the water was calculated as the reference spectrum. Then, the frequency dependent attenuation curve was calculated as the logarithm of the division of the reference spectrum by the spectrum of the signal transmitting though the calcaneus, and BUA was the slope of the curve. Next, in order to eliminate the dependence of BUA on sample thickness [21-23], the normalized broadband ultrasound attenuation (nBUA, dB·MHz^-1^·cm^-1^) with respect to the width of the calcaneus was calculated by:

(2) $$nBUA=\frac{BUA}{l}\text{,}$$

and stiffness index (STI) was calculated by:

(3) STI=0.67×BUA+0.28×SOS−420

For each subject, 8 QUS parameters were measured, with 4 measured with loading (l_SOS, l_BUA, l_nBUA and l_STI, where ‘l_’ means loading) and 4 measured without loading (wl_SOS, wl_BUA, wl_nBUA and wl_STI, where ‘wl_’ means without loading), where the value of each parameter was the average of 5 repetitive measurements. Then, 4 QUS parameters measured under loading condition were normalized by the weight of each subject. Weight-normalized QUS parameters (wn_SOS, wn_BUA, wn_nBUA and wn_STI, where ‘wn_’ means weight-normalized) were calculated by:

(4) $$wn\_Parameter=\frac{l\_Parameter}{weight}$$

### Statistical analysis

The precisions characteristics were estimated from triplicate measurements of 30 subjects. Coefficient of variation (CV %) were 1.53%, 0.24% and 1.87% for nBUA, SOS and STI, respectively. Absolute precision error (root mean square standard deviation: RMS-SD) were 0.980 dB·MHz^-1^·cm^-1^, 3.807 m·s^-1^ and 1.306 for nBUA, SOS and STI, respectively.

Statistical analyses were performed using SPSS Statistics 17.0 (SPSS Inc., Chicago, IL USA). All data were represented in terms of mean ± standard deviation (SD), unless indicated otherwise. Independent-Samples T-test was used to compare the results measured on the left and right calcanea. The Paired-Samples T-test was used to judge the differences between QUS parameters measured with and without loading. One-Way analysis of variance (One-way ANOVA) was used to judge the differences among groups. In order to evaluate the changes of QUS parameters after menopause, the relative changes of mean QUS parameters obtained in the postmenopausal groups (group 1–3) with respect to those obtained in the control group were calculated. The relative changes of mean QUS parameters were calculated by:

(5) $$\Delta X=\frac{\bar{X_{\mathrm{control}}}-\bar{X_{i}}}{\bar{X_{\mathrm{control}}}}\times 100\%\text{,}$$

where ‘*ΔX*’ indicates the relative change of parameter ‘*X*’; ‘$\bar{X_{\mathrm{control}}}$’ means the mean value of ‘*X*’ in the control group; and ‘$\bar{X_{i}}$’ indicates the mean value of ‘*X*’ in the *i*^*th*^ group (*i* = 1, 2, 3).

Because there is consistently high and direct correlation between menopause and osteoporosis [4,12,19], the postmenopausal women are considered as approximations of osteoporosis or osteopenia in this study. Then, the T scores of QUS parameters measured with loading and the weight-normalized QUS parameters for each subject were calculated by:

(6) $$T=\frac{X_{\mathrm{subject}}-\bar{X_{\mathrm{control}}}}{SD_{\mathrm{control}}}\text{,}$$

where ‘*T*’ indicates the T score of parameter ‘X’, ‘$\bar{X_{\mathrm{control}}}$’ means the mean value of ‘*X*’ in the control group (a young healthy population) and ‘$SD_{\mathrm{control}}$’ indicates the standard deviation of ‘$\bar{X_{\mathrm{control}}}$’. Please note that T scores calculated here are different from Z scores which are calculated also by equation (6) but values of the patient's age-matched population are used as the reference values instead. Then, a receiver operating characteristic (ROC) analysis was performed to investigate the discriminatory ability of QUS parameters for menopause.

## Results

**Figure 2:**
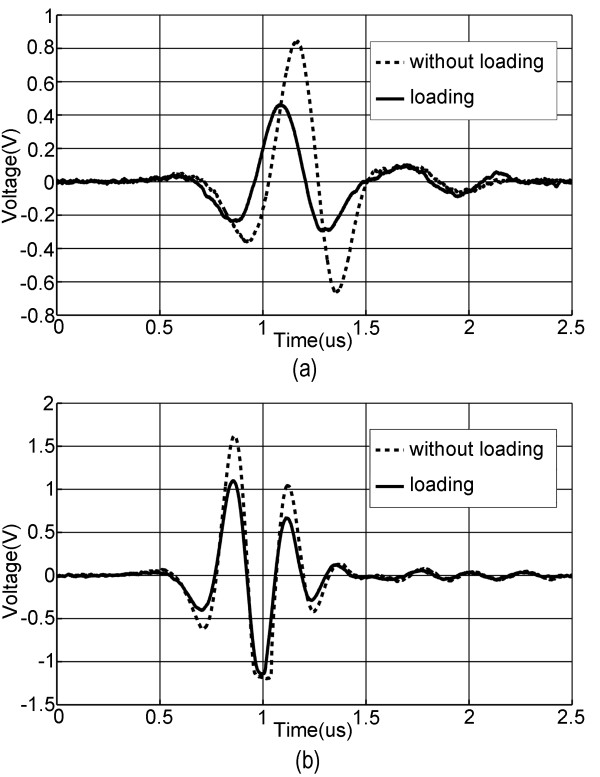
**Typical through transmission ultrasound signals at the calcaneus measured respectively on pre and postmenopausal women with and without loading.** The dash lines indicate the ultrasound signal obtained without loading exerted on calcaneus while the solid lines indicate the ultrasound signal obtained with loading exerted on calcaneus. **(a)** Ultrasound signals transmitting through the calcaneus measured on a typical premenopausal subject with and without loading. **(b)** Ultrasound signals transmitting through the calcaneus measured on a typical postmenopausal subject with and without loading.

Figure 2 shows the typical time-domain ultrasound signals detected by the ultrasound receiver after they transmit through the calcaneus. In this figure, the received ultrasound signals measured with and without loading were compared, on a typical premenopausal female in Figure 2 (a) and a typical postmenopausal female in Figure 2 (b), respectively. It is shown that for both pre and postmenopausal women, the measured ultrasound signals changed when the measuring situation switched from no loading to loading condition, and such changes were different between pre and postmenopausal women.

Independent-Samples T-test revealed no difference between the results measured on the left and right calcanea (*p* = 0.05). The corresponding quantitative data of QUS parameters measured on different subject groups are listed in Table 1. All parameters in Table 1 showed negative relationships with YSM. One-way ANOVA indicated differences on all QUS parameters between the control group and other three postmenopausal groups both under loading and no loading conditions (*p* < 0.05), and differences on wl_nBUA, l_nBUA and wl_STI between group 1 and 3 (*p* < 0.05).

**Table 1:** QUS parameters measured among different subject groups

	**Group 0 (control)**	**Group 1**	**Group 2**	**Group 3**
With loading				
SOS (m·s^-1^)	1596.84 ± 33.16	1570.24 ± 36.98^a^	1566.80 ± 32.60^a^	1553.51 ± 18.83^a^
nBUA (dB·MHz^-1^·cm^-1^)	19.66 ± 4.90	15.22 ± 3.18^b^	13.95 ± 5.87^b^	11.72 ± 3.19^b,c^
STI	86.08 ± 19.44	70.12 ± 16.69^a^	65.32 ± 22.19^a^	55.65 ± 14.24^a^
Without loading				
SOS (m·s^-1^)	1596.67 ± 30.18	1574.07 ± 35.83^a^	1568.63 ± 31.77^a^	1558.36 ± 15.60^a^
nBUA (dB·MHz^-1^·cm^-1^)	18.65 ± 4.18	14.57 ± 2.92^b^	12.97 ± 5.45^b^	11.25 ± 3.07^b,c^
STI	83.21 ± 16.66	69.14 ± 15.96^b^	68.94 ± 14.76^b^	52.75 ± 9.66^b,c^

^a^ Significantly different from group 0 (*p* < 0.05).

^b^ Significantly different from group 0 (*p* < 0.005).

^c^ significantly different from group 1 (*p* < 0.005).

Table 2 shows the results of weight-normalized QUS parameters. Weight-normalized QUS parameters in postmenopausal groups were significantly lower than those in the control group (*p* < 0.001). Besides, wn_nBUA in group 3 was significantly lower than that in group 1 (*p* < 0.05). In postmenopausal groups, wn_nBUA and wn_STI were negatively related to YSM, while wn_SOS was positively related to YSM.

**Table 2:** The results of weight-normalized QUS parameters

	**wn_SOS** **(m·s**^**-1**^**·kg**^**-1**^**)**	**wn_nBUA** **(dB·MHz**^**-1**^**·cm**^**-1**^**·kg**^**-1**^**)**	**wn_STI**
Group 0	32.13 ± 3.10	0.39 ± 0.10	1.72 ± 0.38
Group 1	25.31 ± 3.43^a^	0.25 ± 0.06^a^	1.13 ± 0.30^a^
Group 2	25.48 ± 3.82^a^	0.23 ± 0.11^a^	1.07 ± 0.42^a^
Group 3	26.58 ± 3.88^a^	0.20 ± 0.05^a,b^	0.94 ± 0.22^a^

The prefix ‘wn_’ means “weight-normalized”

^a^ Significantly different from group 0 (*p* < 0.001).

^b^ significantly different from group 1 (*p* < 0.05).

Table 3 shows the results of Paired-Samples T-test on the differences between QUS parameters obtained with and without loading. In all groups, wl_nBUA was significantly different from l_nBUA (*p* < 0.05). In group 1 and group 3, wl_SOS was significantly different from l_SOS (*p* < 0.05). There was only significant difference between wl_STI and l_STI in the control group (*p* < 0.05).

**Table 3:** **The differences between QUS parameters obtained with and without loading ( *P* values of Paired-Samples T-test)**

**Paired-Samples**	**Group 0**	**Group 1**	**Group 2**	**Group 3**
wl_nBUA Vs. l_nBUA	*p* = 0.002^a^	*p* = 0.007^a^	*p* = 0.001^a^	*p* = 0.009^a^
wl_SOS Vs. l_SOS	*p* = 0.899	*p* = 0.024^a^	*p* = 0.210	*p* < 0.001^a^
wl_STI Vs. l_STI	*p* = 0.017^a^	*p* = 0.270	*p* = 0.296	*p* = 0.126

The prefixes ‘l_’ and ‘wl_’ mean “loading” and “without loading”, respectively

^a^ There is significant difference between Paired-Samples.

The relative changes on mean of different QUS parameters after menopause were calculated by equation (5) and were shown in Table 4. The relative changes of mean QUS parameters measured with loading were higher than those measured without loading (except ΔnBUA in group2 and ΔSTI in group 3); while the relative changes of the weight-normalized QUS parameters after menopause were much higher than those measured without loading.

**Table 4:** Relative changes of mean QUS parameters in postmenopausal groups with respect to control group (%)

		**Group 0**	**Group 1**	**Group 2**	**Group 3**
relative changes of mean speed of sound					
	Δwl_SOS	—	1.42	1.76	2.40
	Δl_SOS	—	1.67	1.88	2.71
	Δwn_SOS	—	21.24	20.71	17.28
relative changes of mean normalized broadband ultrasound attenuation					
	Δwl_nBUA	—	21.88	30.46	39.68
	Δl_nBUA	—	22.58	29.04	40.39
	Δwn_nBUA	—	35.90	41.03	48.72
relative changes of mean stiffness index					
	Δwl_STI	—	16.91	17.15	36.61
	Δl_STI	—	18.54	24.12	35.35
	Δwn_STI	—	34.30	37.79	45.35

The prefixes ‘l_’, ‘wl_’ and ‘wn_’ mean “loading”, “without loading” and “weight-normalized”, respectively.

The area under the curve (AUC) showed the ability of each parameter to discriminate postmenopausal women (see Table 5). The AUCs of QUS parameters measured without loading were little higher than, but not significantly different from, the AUCs of their counterparts measured with loading. However, AUCs increased significantly when weight-normalized QUS parameters were used, and the AUCs of all weight-normalized QUS parameters were higher than 0.9.

**Table 5:** The discriminatory ability of QUS parameters for menopause

**Parameter**	**AUC**	**Std. Error**	** *P* **	**Asymptotic 95% CI**
wl_nBUA	0.831	0.042	<0.001	0.749 - 0.912
l_nBUA	0.818	0.043	<0.001	0.733 - 0.903
wn_nBUA	0.922	0.027	<0.001	0.868 - 0.975
wl_SOS	0.845	0.042	<0.001	0.763 - 0.926
l_SOS	0.843	0.042	<0.001	0.761 - 0.926
wn_SOS	0.905	0.026	<0.001	0.854 - 0.956
wl_STI	0.809	0.046	<0.001	0.719 - 0.899
l_STI	0.798	0.045	<0.001	0.710 - 0.887
wn_STI	0.914	0.029	<0.001	0.856 - 0.971

The prefixes ‘l_’, ‘wl_’ and ‘wn_’ mean “loading”, “without loading” and “weight-normalized”, respectively

AUC: area under the curve; CI: confidence interval.

## Discussion

The main finding of our study is the self-weight loading mainly influences the ultrasound attenuation in calcaneus QUS measurement. And the loading-induced changes on QUS parameters were more on postmenopausal women than those on premenopausal women. The results of this study indicated that calcaneus QUS measurement performed with loading may be a probable way to improve the QUS based osteoporosis diagnosis.

It is well-known that the BMD measured by DXA is the golden standard for osteoporosis diagnosis. However, in this study we did not use the BMD as the reference indicator of osteoporosis. The reason why we did so are two: firstly, we consider the radiation of DXA measurement may do harm to the subjects; secondly, this is a preliminary study in which we just want to address the potential but not to draw the conclusion whether adding a physical loading can improve the QUS based osteoporosis diagnosis. Therefore, considering the high and direct correlation between menopause and osteoporosis, we used the postmenopausal women as approximations of osteoporosis or osteopenia in this study when DXA measurement was not performed. Moreover, we understand that further tests using DXA measurement as diagnosis reference must be performed in proceeding study to conclude whether adding a physical loading can improve the QUS based osteoporosis diagnosis.

Since trabecular bone is an inhomogeneous porous medium, the interaction between physical loading and bone and the interaction between ultrasound and bone are highly complex phenomenon. Modelling ultrasonic propagation through trabecular bone tissue using porous media theories showed that the sound velocity and attenuation in trabecular bone depend on the frequency of the ultrasound, the elastic properties of the constituting materials, porosity, permeability, tortuosity, and effective stress [15]. Previous studies also suggested that QUS parameters were highly associated with bone microstructure and mechanical properties [15-18]. While a loading is exerted on bone, the bone trabecula buckle and bend, and a strain of the bone takes place. The deformation of the bone may change the microstructure and some other properties of the bone (e.g. the porosity and permeability), resulting in the changes of reflection and scattering of the ultrasound signal transmitting through the bone, thus the ultrasound property parameters change accordingly.

Paired sample T-test was used to determine whether there were significant differences between pairs of values measured on the same subject under two different loading conditions. The testing results (shown in Table 3) indicated that QUS parameters, mainly nBUA, significantly changed when loading was exerted on calcaneus. The nBUAs obtained with and without loading were different in all subject groups. We presumed that the deformation of the bone microstructure under self-weight loading is the main reason for these differences. As pointed out by Haiat et al [24], nBUA showed strong correlations with the ratio of bone volume to tissue volume (BV/TV) that 20% change of BV/TV resulted in a change of nBUA by 45 dB·MHz^-1^·cm^-1^. In our study, the propagation path of the ultrasound beam can be regarded as a cylinder of bone tissue. Since the diameter of the transducers (i.e. the diameter of the cross section of the cylinder) and the width of the calcaneus (i.e. the height of the cylinder) are constants, the volume of the cylinder is a constant (i.e. the tissue volume (TV) is a constant) either with or without loading. The calcaneus trabecula however buckle and bend when loading is applied, and these deformations lead to the strain of bone. The strain induced by loading will reduce the space among trabecula, compress more bone tissue into the ultrasound path, increase the value of BV, and then increase the value of BV/TV. And the ultrasound attenuation increased correspondingly.

It was found in our results that the sensitivities of SOS and STI to loading were lower than that of nBUA. There were only statistically significant differences between SOS measured with and without loading in group 1 and 3. Different behaviours of SOS and nBUA to loading may probably result from the fact that nBUA is mainly influenced by the microstructure of the bone while SOS is mainly determined by bone density [7-10]. Self-weight loading causes deformations on the bone (deformation of microstructure); however, it does not alter the density and elasticity of the bone. In addition, as a combination of BUA and SOS, STI measured with loading only differed from that measured without loading in the control group. One possible reason is that STI was calculated from an empirical equation (equation (3)) based on the experimental results measured without loading and this equation may be no longer suitable for calculating STI under loading condition.

Our results showed that some QUS parameters, mainly nBUA, substantially changed if loading was exerted on bone during measurement. Then, it should be validated that whether the changes of QUS parameters induced by loading influence the QUS based osteoporosis diagnosis or not. The experimental results reported by Wright et al [25] may provide some hints to us. According to their study, under the same loading stress, the strain of healthy bone was much lower than that of the decalcified and deproteinized bones. Since the calcium and protein in the osteoporotic bone are lower than those in the healthy bone, the strain of healthy bone should be lower than that of the osteoporotic bone under the same loading condition. That results in smaller deformations on the microstructure of the healthy bone than that of the osteoporotic bone. The resultant changes of QUS parameters of the healthy bone should be smaller than those of the osteoporotic bone. As a result, the differences on QUS parameters between healthy and osteoporotic bones should be enlarged when the QUS measurement is performed with the same loading exerted on the bone.

The analyses above can be well validated by the data shown in Table 4. Although the relative changes of QUS parameters in postmenopausal women with respect to healthy women under loading condition were similar to those measured without loading, the relative changes of weight-normalized QUS parameters (i.e. with loading and all subjects subject to approximately the same stress -1kg stress) were much higher than those of the QUS parameters obtained without loading. These results indicated that the differences between pre and postmenopausal women were larger when weight-normalized QUS parameters were used.

In ROC analysis, the AUCs of QUS parameters measured with loading were not higher (even a few lower) than those measured without loading. However, after corresponding QUS parameters being normalized by weight, the AUCs increased significantly (higher than 0.9) from their counterparts obtained from QUS parameters measured without loading. This meant that only the QUS parameters measured under the same amount of physical loading showed stronger discriminatory ability for menopause in this study. On another word, weight-normalized QUS parameters eliminated the variations of weight induced loadings which exerted on different subjects, and revealed the true effect of physical loading on ultrasound measurement. The results of ROC analysis suggested that the discriminatory ability of weight-normalized QUS parameters for menopause was stronger than that of traditional QUS parameters.

To our best knowledge, this is the first study that investigated the effect of loading on the bone QUS measurement and further proposed the weight-normalized QUS parameters. The weight-normalized QUS parameters were proposed by exerting the physiological loading on traditional QUS parameters. In this study, weight-normalized QUS parameters were significantly lower in postmenopausal groups than those in the control group and the relative changes of the weight-normalized QUS parameters after menopause were much higher than those measured without loading. It is worth noting that there is more weight in subjects in postmenopausal groups than those in the premenopausal group in this study. However, this fact cannot be the main reason for the results above, since previous studies [26,27] have shown that QUS parameters, including SOS, nBUA and STI, were in positive correlation to weight. It means that more weight companioned with higher values of QUS parameters. Meanwhile, the mean weight of group 3 was the lowest in postmenopausal groups, but the corresponding mean value of weight-normalized nBUA and weight-normalized STI were the lowest and the relative changes of weight-normalized nBUA and weight-normalized STI were the highest in postmenopausal groups. So the lower values and the higher relative changes of weight-normalized QUS parameters in postmenopausal groups cannot be simply ascribed to more weight in subjects in postmenopausal groups.

In this study, the effect of loading was adopted as an initial attempt to bone QUS measurement. Although there are some limitations on these parameters, our results suggested that weigh-normalized QUS parameters could discriminate pre and postmenopausal women more effectively. And by considering the high relationship between menopause and osteoporosis, weight-normalized QUS parameters showed its potential advantages on osteoporosis diagnosis and further investigations about how much stress exerted on the bones is suitable for this method and how to exert the same stress on different subjects are needed.

## Conclusions

QUS parameters (mainly nBUA) measured on the calcaneus significantly changed after loading was exerted during QUS measurement. Between pre and postmenopausal women, the differences on weight-normalized QUS parameters were larger than those on traditional QUS parameters. Weight-normalized QUS parameters measured under loading condition can discriminate postmenopausal women from premenopausal women more effectively. This study reveals that loading is an important factor which mainly influences bone ultrasound attenuation, and furthermore, we infer that adding physical loading during QUS measurement may be a probable way to improve the QUS based osteoporosis diagnosis. Further studies will be conducted next to reveal the mechanism of physical loading on QUS measurement.

## Competing interests

The authors declare that they have no competing interests.

## Authors' contributions

CR L participated in the design of the study, data collection, analysis of the data, and drafting the manuscript. HJ N participated in design of the study and drafting the manuscript. F P participated in design of the study. L W participated in drafting the manuscript. LW S participated in the design of the study. YB F critically commented upon the study. DY L participated in the design of the study, analysis of the data, and drafting the manuscript. All authors read and approved the final manuscript.

## Pre-publication history

The pre-publication history for this paper can be accessed here:

http://www.biomedcentral.com/1471-2474/13/70/prepub
